# Development of chromosome-specific markers with high polymorphism for allotetraploid cotton based on genome-wide characterization of simple sequence repeats in diploid cottons (*Gossypium arboreum* L. and *Gossypium raimondii* Ulbrich)

**DOI:** 10.1186/s12864-015-1265-2

**Published:** 2015-02-06

**Authors:** Cairui Lu, Changsong Zou, Youping Zhang, Daoqian Yu, Hailiang Cheng, Pengfei Jiang, Wencui Yang, Qiaolian Wang, Xiaoxu Feng, Mtawa Andrew Prosper, Xiaoping Guo, Guoli Song

**Affiliations:** State Key Laboratory of Cotton Biology, Institute of Cotton Research of Chinese Academy of Agricultural Sciences, Anyang, 455000 China; National Key Laboratory of Crop Genetic Improvement, Huazhong Agricultural University, Wuhan, 430070 China

**Keywords:** Chromosome-specific, SSR, Tetraploid cotton, Genome-wide

## Abstract

**Background:**

Tetraploid cotton contains two sets of homologous chromosomes, the At- and Dt-subgenomes. Consequently, many markers in cotton were mapped to multiple positions during linkage genetic map construction, posing a challenge to anchoring linkage groups and mapping economically-important genes to particular chromosomes. Chromosome-specific markers could solve this problem. Recently, the genomes of two diploid species were sequenced whose progenitors were putative contributors of the At- and Dt-subgenomes to tetraploid cotton. These sequences provide a powerful tool for developing chromosome-specific markers given the high level of synteny among tetraploid and diploid cotton genomes. In this study, simple sequence repeats (SSRs) on each chromosome in the two diploid genomes were characterized. Chromosome-specific SSRs were developed by comparative analysis and proved to distinguish chromosomes.

**Results:**

A total of 200,744 and 142,409 SSRs were detected on the 13 chromosomes of *Gossypium arboreum* L. and *Gossypium raimondii* Ulbrich, respectively. Chromosome-specific SSRs were obtained by comparing SSR flanking sequences from each chromosome with those from the other 25 chromosomes. The average was 7,996 per chromosome. To confirm their chromosome specificity, these SSRs were used to distinguish two homologous chromosomes in tetraploid cotton through linkage group construction. The chromosome-specific SSRs and previously-reported chromosome markers were grouped together, and no marker mapped to another homologous chromosome, proving that the chromosome-specific SSRs were unique and could distinguish homologous chromosomes in tetraploid cotton. Because longer dinucleotide AT-rich repeats were the most polymorphic in previous reports, the SSRs on each chromosome were sorted by motif type and repeat length for convenient selection. The primer sequences of all chromosome-specific SSRs were also made publicly available.

**Conclusion:**

Chromosome-specific SSRs are efficient tools for chromosome identification by anchoring linkage groups to particular chromosomes during genetic mapping and are especially useful in mapping of qualitative-trait genes or quantitative trait loci with just a few markers. The SSRs reported here will facilitate a number of genetic and genomic studies in cotton, including construction of high-density genetic maps, positional gene cloning, fingerprinting, and genetic diversity and comparative evolutionary analyses among *Gossypium* species.

**Electronic supplementary material:**

The online version of this article (doi:10.1186/s12864-015-1265-2) contains supplementary material, which is available to authorized users.

## Background

Simple sequence repeats (SSRs) or microsatellites are short (often defined as 1–6 base pairs (bp)) tandem repeat nucleotides in DNA sequences. They are ubiquitous in genomes, being found in both eukaryotes and prokaryotes and in any region (protein coding and non-coding) [1,2]. Because of their high mutation rates via insertion or deletion of one or a few repeat units, SSRs have been developed into one of the most popular sources of codominant markers with high information content during the past several years [3]. They are widely employed in many research areas, including linkage mapping, population genetics, phylogenetics, and comparative genomics [4-8]. Additionally, recent studies reveal that microsatellites may serve an important role in regulating gene expression, protein function, and genome evolution [9-12]. The conventional strategies of developing SSR markers are screening genomic DNA libraries or constructing SSR-enriched libraries [13]. Both are usually time-consuming and labor-intensive. However, the availability of genome sequences allows us to mine for SSRs at the genomic level *in silico*, and analysis of these SSRs has provided insight into their distributions, putative functions, and evolution [14,15].

Cottons (Malvaceae: *Gossypium*) comprise five tetraploid (2n = 52) and over 45 diploid (2n = 26) species [16]. The diploid species are grouped into eight subgenomes, designated A–G and K [17,18]. The A-genome (1,687 Mb/1C) is almost twice the size of the D-genome (880 Mb/1C) [19]. Tetraploid cotton species are thought to have formed through hybridization and subsequent polyploidization between an A-genome and a D-genome species [20]. Analyses of genetic maps revealed that diploid A- and D-genomes have a high level of synteny with At- and Dt-genomes in tetraploid cotton [21-25].

The tetraploid *G. hirsutum,* also known as upland cotton, produces over 90% of the world’s cotton [26]. However, because of the duplicated DNA segments and homologous chromosomes in its tetraploid genome, many markers map to at least two positions [20-23,27-32], most on homologous chromosomes, hindering the precise mapping of qualitative character genes or quantitative trait loci (QTLs) to particular chromosomes. Furthermore, given the narrow genetic base, intraspecific genetic diversity in cottons is relatively low (~5%), both among and within *G. hirsutum* cultivars [33-38], hindering the development of high-resolution genetic maps and marker-assisted selection (MAS) breeding in cottons. However, chromosome-specific markers with high polymorphism distributed throughout the genome would facilitate chromosome identification in genetic maps of tetraploid cottons, which are instrumental for applications like positional gene cloning, especially in discriminating homologous chromosomes during fine mapping of single traits and QTLs.

Traditional methods including meiotic fluorescence in situ hybridization (FISH) and translocation lines can be used to precisely locate DNA markers to chromosomes, but most markers are too small to be used directly as probes in FISH. Bacterial artificial chromosome (BAC) clones were often used as chromosome-specific markers [39-41], but the process is time-consuming and labor-intensive, and only a few BACs have been identified as chromosome-specific [42,43]. Large-scale development of chromosome-specific SSRs can be realized only when each chromosome sequence of the cotton genome is obtained. Recently, from the whole-genome sequence of *G. raimondii* [44], Zou et al. [45] identified 136,345 microsatellites, and 112,177 primer pairs were designed, but they are insufficient for mining chromosome-specific SSRs when only one progenitor genome sequence is present. The availability of a very large set of chromosome-specific SSRs distributed throughout the genome would benefit the cotton research and breeding community.

The rapid development of high-throughput DNA sequencing technologies have allowed increasing numbers of genomes to be sequenced, especially multiple genomes within a genus, expediting the development of chromosome-specific markers and detailed comparative mapping. In recent years, the genomes of *G. raimondii* [44,46] and *G. arboreum* [47] (both genome assembly and annotation are available at http://cgp.genomics.org.cn) have been sequenced; the progenitors of these two diploid species were putative contributors of the A- and D-subgenomes to tetraploid cotton. These diploid genomes share a high level of synteny or co-linearity with the subgenomes in tetraploid cotton [20-23,25]. In spite of the importance of chromosome-specific microsatellite markers to many applications, their systematic and genome-wide characterization in cotton genomes has not yet be conducted. In the present study, we characterized the distribution and density of microsatellites in *G. raimondii* and *G. arboreum* chromosomes and identified chromosome-specific SSRs by comparative analysis. In addition, a set of highly-polymorphic SSRs were selected to construct a genetic linkage map of two homologous chromosomes (chr07 and chr16) in tetraploid cotton, proving that chromosome-specific SSRs could distinguish homologous chromosomes during genetic mapping.

## Results

We analyzed the distributions of SSRs with ≥ 3 repeat units and a minimum total length of 15 bp in each of the 13 chromosomes sequences of *G. arboreum* (downloaded from http://cgp.genomics.org.cn) and *G. raimondii* (downloaded from NCBI) to understand their general features in cotton genomes. SSRs on each chromosome were then compared those on the other 25 chromosomes (12 in the same genome and 13 in the other genome) to find chromosome-specific SSRs. Standardized motifs used in SciRoKo [48] were used to represent all variants on both strands of the DNA sequence (e.g., AG also includes GA and the reverse complements CT and TC) for consistency in estimating repeat frequencies. Unless otherwise specified, SSR content was expressed as ‘number of SSRs per million base pairs’ or as relative frequencies (%) within a particular dataset. Using the chromosome-specific SSRs, we constructed two linkage groups for two homologous chromosomes (chr07 and chr16) in the tetraploid cotton genome to test whether these chromosome-specific SSRs could distinguish homologous chromosomes.

### Distribution of SSR types in genomes and on individual chromosomes

The SSR content of the *G. arboreum* and *G. raimondii* genomic sequences are summarized in Table 1. A total of 200,744 SSRs were detected from 13 *G. arboreum* chromosome sequences, giving an overall density across the chromosomes of 131.03 SSRs/Mb (one SSR every 7.63 kb; Additional file 1). 142,409 SSRs were detected in the 13 *G. raimondii* chromosome sequences, giving an overall density of 190.08 SSRs/Mb (one SSR every 5.26 kb; Additional file 2). Although the *G. arboreum* genome is about twice the size of the *G. raimondii* genome the SSR content did not exhibit a two-fold difference; *G. raimondii* had a much higher SSR density (190.08 SSRs/Mb) than *G. arboreum* (131.03 SSRs/Mb). To enable convenient and selective use of the newly developed SSRs, a BLAST search to determine redundancy was performed against publically available SSRs in the CottonGen database (www.cottongen.org); 38,044 SSRs were recognized as duplicates (Additional files 1 and 2).

**Table 1:** **Distribution of SSRs with ≥ 3 repeats and a minimum length of 15 bp in genomic sequences of** ***Gossypium arboreum*** **and** ***Gossypium raimondii***

**Sequence type**	G. arboreum*				G. raimondii*			
	**Count**	**Rel. freq. (%)**	**Mean repeat number**	**Density (SSR/Mb)**	**Count**	**Rel. freq. (%)**	**Mean repeat number**	**Density (SSR/Mb)**
Mononucleotide	20,112	10.02	18.89	13.13	9812	6.89	17.47	13.10
Dinucleotide	44,204	22.02	11.38	28.85	28168	19.78	12.03	37.60
Trinucleotide	29,914	14.90	10.20	19.53	21044	14.78	8.80	28.09
Tetranucleotide	28,341	14.12	5.03	18.50	25425	17.85	4.93	33.93
Pentanucleotide	58,078	28.93	3.68	37.91	42546	29.86	3.69	56.79
Hexanucleotide	20,095	10.01	4.09	13.12	15414	10.82	3.92	20.57
Total/mean	200,744	100	8.88	131.03	142409	100	8.47	190.08
Total seq (Mbp)	1532				749			

*Only SSRs anchored in chromosomes were considered.

**Figure 1:**
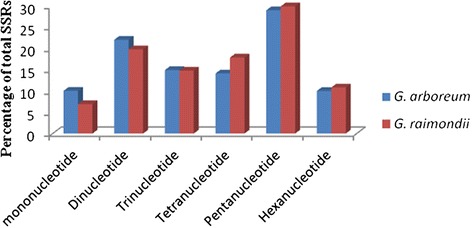
**Relative frequency (%) of SSR types in the genomes of** ***Gossypium arboreum*** **and** ***Gossypium raimondii*** **.** SSRs are organized by the number of repeats. The graph was based on a total of 200,744 and 142,409 SSRs detected in the genomes of *G. arboreum* and *G. raimondii*, respectively.

Pentanucleotide repeats were the most common SSR type in both *G. arboreum* and *G. raimondii,* representing 28.93% and 29.86 of all SSRs, respectively, followed by dinucleotide repeats (22.02% and 19.78%; Figure 1 and Table 1). No major differences were observed between *G. arboreum* and *G. raimondii*, aside from the higher density of SSRs in *G. raimondii*. The distribution of SSR types in the two cotton genomes differed from those of cucumber, poplar and grapevine, in which tetranucleotide repeats were the most frequent type, and also from those of rice and *Arabidopsis*, in which trinucleotide repeats were most frequent [49-51].

The SSR contents of each chromosome sequence in *G. arboreum* and *G. raimondii* are summarized in Table 2. In general, no significant differences were detected among chromosomes within a genome in the distribution of SSRs, except that chr02 in *G. arboreum* had a relative low density. For each chromosome, the distribution of motifs was consistent with their genome features, pentanucleotides were the most common SSR type followed by dinucleotides in every chromosome, and no motifs were enriched on particular chromosomes. The variation in SSR density among chromosomes was larger in *G. arboreum* (range: 107.1–151.7 SSRs/Mb) than in *G. raimondii* (177.6–204.9 SSRs/Mb; Additional file 3).

**Table 2:** **Distribution of total and chromosome-specific SSRs among chromosome sequences of** ***Gossypium arboreum*** **and** ***Gossypium raimondii***

**Chromosome**	***G. arboreum***				***G. raimondii***			
	**Total SSR No.**	**Density (SSR/Mb)**	**Chromosome-specific No.**	**Rel. freq. (%)**	**Total SSR No.**	**Density (SSR/Mb)**	**Chromosome-specific No.**	**Rel. freq. (%)**
Chr01	18522	125.89	10696	57.75	10831	193.86	7157	66.08
Chr02	10820	107.1	5689	52.58	11366	181.07	7403	65.13
Chr03	17172	134.45	9915	57.74	8395	183.44	4422	52.67
Chr04	13698	133.07	7788	56.86	11677	187.8	7634	65.38
Chr05	8653	144.96	5191	59.99	11393	177.63	7505	65.87
Chr06	15070	142.58	8974	59.55	9816	192.19	6454	65.75
Chr07	18077	131.3	10504	58.11	12495	204.9	8329	66.66
Chr08	16801	138.47	9609	57.19	11006	192.65	7112	64.62
Chr09	17399	151.72	10242	58.87	14444	204.26	9602	66.48
Chr10	15306	120.15	8560	55.93	11119	178.84	7272	65.40
Chr11	13863	143.49	8031	57.93	11696	186.59	7872	67.31
Chr12	17524	120.03	9651	55.07	7018	198.08	4698	66.94
Chr13	17839	123.75	10245	57.43	11153	191.23	7344	65.85
Total/mean	200,744	132.07	115095	57.31	142409	190.20	92804	64.93

### Distribution of SSR motifs

**Figure 2:**
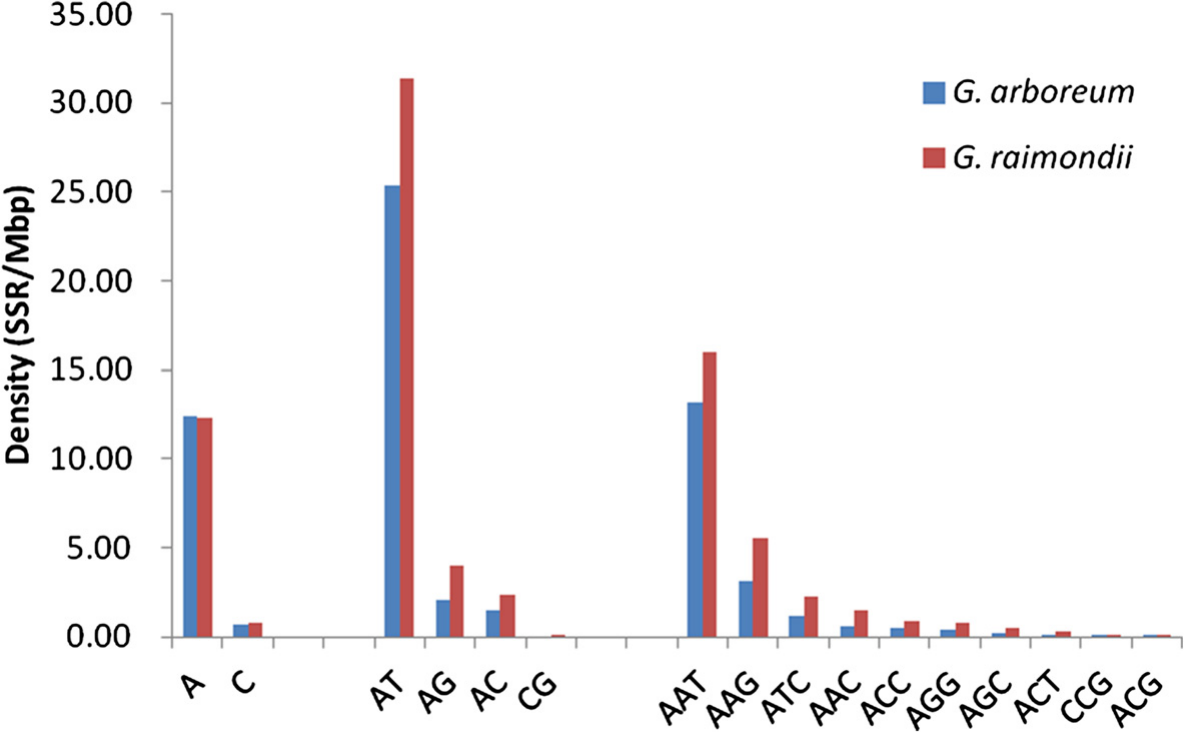
**Distribution of di- and trinucleotide repeats in the genomes of** ***Gossypium arboreum*** **and** ***Gossypium raimondii*** **.** Frequency values are expressed as the number of repeats per million base pairs of sequence. Detailed information on frequencies of individual di- and trinucleotide repeat motifs is provided in Additional file 2.

Detailed analyses of individual repeat motifs were carried out for each type of SSR found in *G. arboreum* and *G. raimondii* (Additional file 4). In general, *G. raimondii* had a higher density of all repeat types except A motifs, which were similarly and significantly overrepresented in both *G. arboreum* (94.5% of mononucleotide motifs) and *G. raimondii* genomes (94.1%; Figure 2 and Additional file 4). Among dinucleotide repeats, the AT motif was dramatically overrepresented in both genomes (Figure 2 and Additional file 4), representing 87.9% of the dinucleotide motifs in *G. arboreum* and 83.3% in *G. raimondii*. AT repeats were also the most abundant motif overall in cotton genomes (25.36 and 31.33 SSRs/Mb), accounting for 19.36% and 16.48% of the total SSRs, respectively. CG repeats were the least frequent dinucleotides; only five were found in *G. arboreum* and 10 in *G. raimondii*.

Among the trinucleotides, repeats of AAT were most common in both the *G. arboreum* (67.6% of trinucleotide motifs) and *G. raimondii* (57.0%) genomes (Figure 2). ACG was the rarest trinucleotide motif, unlike in most dicot plants in which CCG is least common (although CCG is the most abundant trinucleotide motif in the monocots rice and sorghum) [51-53]. Among tetranucleotide repeats, the AT-rich motifs AAAT, AAAG, AATT, and ATAC were, in that order, the most abundant in both genomes, together representing 86.2% of all tetramer repeats in *G. arboreum* and 85.0% in *G. raimondii*. GC-rich repeats, like CCCG, ACGG and ACCG, were rarest, with relative frequencies of about 0.1% (Additional file 4). The most abundant motif AAAT had nearly twice the density in *G. raimondii* than in *G. arboreum*. As with the dinucleotides and other kinds of motif, the density in *G. raimondii* was slightly higher than in *G. arboreum*.

Among pentanucleotide repeats, the AT-rich motifs AAAAT, AAAAG, and AAATT were most frequent in both genomes, together accounting for 65.11% of all pentanucleotides in *G. arboreum* and 55.33% in *G. raimondii* (Additional file 4). AAAAT was the most abundant, outnumbering the next most frequent repeats, AAAAG or AAATT, by about three fold. Surprisingly, although GC-rich repeats like CCGCG, CCCCC and ACCGT were the rarest, CCCGG motif was relatively abundant in both genomes. Among hexanucleotides, AT-rich motifs also predominated in both genomes; AAAAAT was the most common hexanucleotide repeat in both genomes, but the next most frequent was AATCAG in *G. arboreum* and AAAAAG in *G. raimondii*.

In general, AT-rich motifs were most common, especially A-rich ones (the most abundant repeats were A, AT, AAT, AAAT, AAAAT, and AAAAAT in their respective size classes). *G. raimondii* had higher density of all repeat types than *G. arboreum* except the A motif (with similar frequencies), while *G. arboreum* had more repeats due to larger genome. For the same SSR type, the genomes tended to accumulate motifs containing more A in tandem repeat. For example, AAATT was more abundant than AATAT in the pentanucleotides, and AAAATT was more abundant than AAATAT in the hexanucleotides.

### Development of chromosome-specific SSRs

We first examined SSR repetition within each chromosome to detect whether a small number of SSR types dominated, given the high repeat content in the cotton genome. Each SSR sequence, including the repeat and both flanking sequences, were compared with all other SSR sequences on the same chromosome. The average proportion of uniSSRs was 70.78% on *G. arboreum* chromosomes and 80.00% on *G. raimondii* chromosomes. Thus, only 20–30% SSRs were repetitive within chromosomes, which is conducive to obtaining chromosome-specific SSRs. For single repetitive SSRs, *G. arboreum* had an average of 3.1 repetitions and *G. raimondii* and average of 2.7. The average maximum number of repetitions was 264 in *G. arboreum* and only 89 in *G. raimondii*, so the former had a higher degree of repetition than the latter (Additional file 5).

SSR sequences from each chromosome were then compared with those on the other 25 chromosomes (Additional file 6); similar SSRs were then excluded to identify chromosome-specific SSRs for each chromosome. The genome of *G. arboreum* had more chromosome-specific SSRs than did *G. raimondii*. The average proportion of chromosome-specific SSRs was higher in *G. raimondii* (64.93%) than in *G. arboreum* (57.31%, Table 2).

As the SSRs were designed using diploid cotton genome sequences, it is important to check the percentage of SSRs that could be successfully amplified in tetraploid cottons. Of 907 successfully designed SSRs, 855 (94.3%) could be amplified in TM-1, Hai 7124, and Liaomian 7. These chromosome-specific SSRs are therefore considered suitable for amplification in tetraploid cotton.

### Chromosome-specific SSRs could distinguish homologous chromosomes in tetraploid cotton

To determine whether the chromosome-specific SSRs could distinguish homologous chromosomes in other tetraploid cotton species, two homologous linkage groups were constructed using 500 SSRs from chr01 of *G. arboreum* and 500 SSRs from chr01 of *G. raimondii*. A total of 459 and 448 primer pairs were designed from *G. arboreum* and *G. raimondii*, respectively.

**Figure 3:**
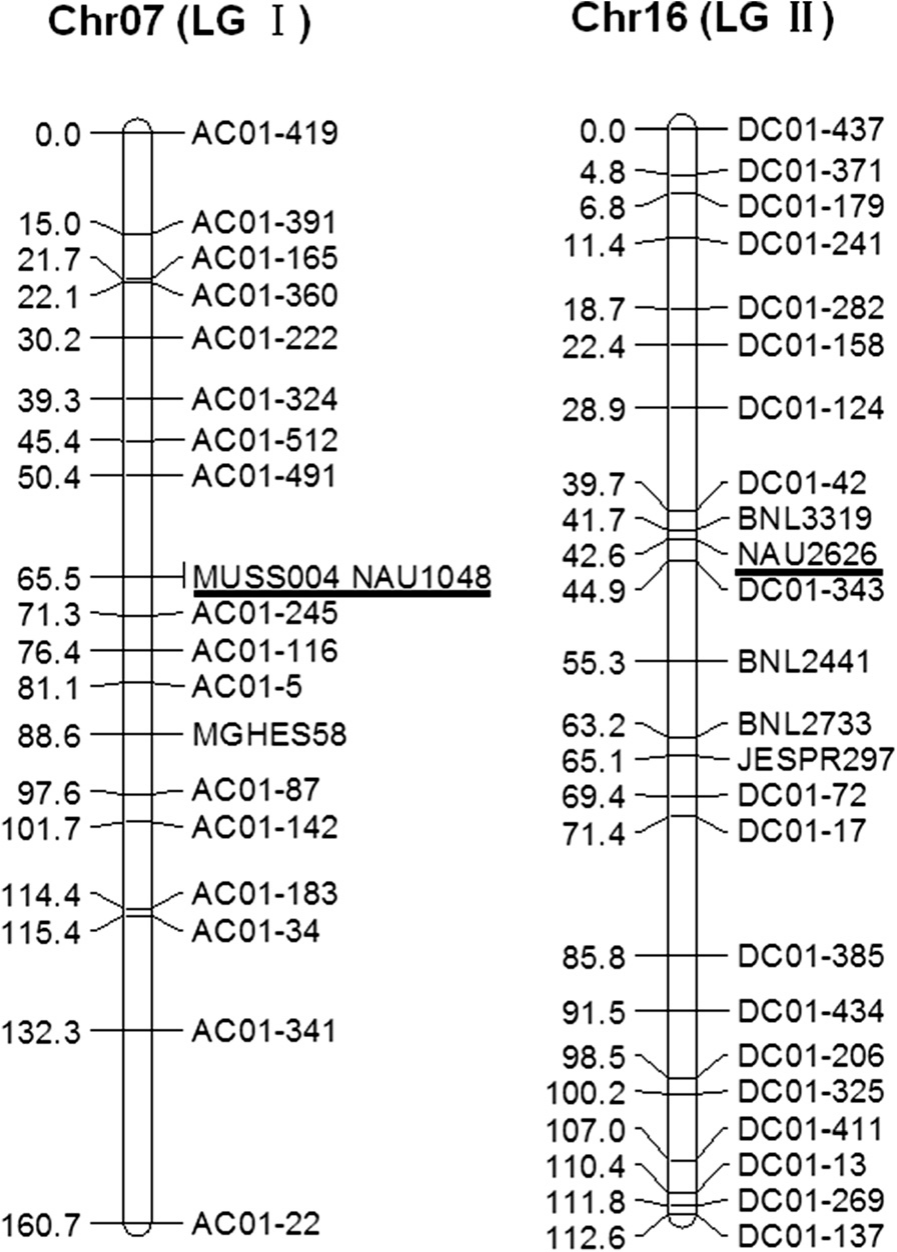
**Genetic linkage groups of chr07 and chr16 in tetraploid cotton.** Linkage groups were developed from 184 individuals of a BC_1_ population derived from TM-1 and T586 using chromosome-specific SSRs. AC01 represents chromosome-specific SSRs designed from chr01 of *Gossypium arboreum* (corresponding to chr07 of tetraploid cotton). DC01 represents chromosome-specific SSRs designed from chr01 of *Gossypium raimondii* (corresponding to chr16 of tetraploid cotton). Markers MUSS004 and NAU1048 mapped only to chr07, and NAU2626 mapped only to chr16 in the published genetic maps. The other published markers, including MGHES58, BNL3319, BNL2441, BNL2733, JESPR297, mapped to one or more other chromosomes, in addition to chr07 or chr16 in the published genetic maps.

A BC_1_ population of 184 individuals was constructed by crossing TM-1 and T586 (both are *G. hirsutum*). 22, 23, and 9 polymorphic markers between the parents were obtained from the 459 SSRs designed from chr01 of *G. arboreum*, 448 from chr01 of *G. raimondii*, and 211 from published genetic maps, respectively. All 54 SSRs were included in the linkage analysis. Two linkage groups, containing 44 markers, were constructed: 20 markers in linkage group (LG) I, and 24 in LG II (Figure 3). The other 10 loci were not part of any linkage group. 17 of the 22 polymorphic SSRs from *G. arboreum* mapped to LG I, and 19 of the 23 SSRs designed from *G. raimondii* mapped to LG II. No SSR designed from *G. arboreum* mapped to LG II, and no SSR designed from *G. raimondii* mapped to LG I.

Three markers designed from published resources mapped to LG I; among them, NAU1048 and MUSS004 only mapped to chr07 in tetraploid cotton in the published maps [28,30,54-59]. Similarly, five markers designed from published work mapped to LG II, including NAU2626, which only mapped to chr16 in tetraploid cotton [28,57,58]. Thus, LG I was designated as being on chr07, and LG II on chr16. Other markers mapped to more than one chromosome in different genetic maps, usually both chr07 and chr16; as an extreme, JESPR297 was mapped on chr07, chr09, chr15, and chr16 in various maps [28,30,31,57,58,60-65]. Clearly, chromosome-specific SSRs designed from diploid cotton genomes could be mapped accurately to corresponding chromosomes in tetraploid cotton.

### High polymorphism of designed chromosome-specific SSRs between upland and sealand cotton inbred lines

High polymorphism is the most important characteristic for molecular markers, even those designed to be chromosome-specific. Although there were several thousand chromosome-specific SSRs it was important to identify the most polymorphic ones for map construction and gene mapping. SSR polymorphism is correlated with repeat length, and dinucleotide AT-rich repeats are more polymorphic than other kinds of repeats [51]. Therefore, the SSRs designed in this study were selected from longer repeats of dinucleotide AT-rich chromosome-specific SSRs. However, the polymorphism rate was almost identical between AT-repeat SSRs and public SSR markers. This is likely due to the near-isogenic relationship of the two parents (TM-1and T586), which limit the polymorphism of all kinds of markers to a very low level. To test whether the AT-repeat SSRs were more polymorphic than normal SSRs, a further upland cotton variety (Liaomian 7) and a sealand cotton variety (Hai 7124) were employed for genotyping against TM-1. Fifty-two percent of the AT-repeat SSRs showed polymorphism between TM-1 and Hai 7124, while 40% of the public SSRs showed polymorphism. A similar situation was observed between the two upland cottons (TM-1 and Liaomian 7): 10% of AT-repeat SSRs showed polymorphism, versus 6% of public SSRs. Therefore, the AT-repeat SSRs selected in this study were deemed more polymorphic than normal SSRs.

## Discussion

### Frequency differences between *G. arboreum* and *G. raimondii*

Genome-wide analysis of SSRs provides valuable information for genetic, genomic, and evolutionary studies. In this study, we analyzed the distributions and frequencies of SSRs with motifs of 1–6 bp and a minimum of three repeat units in two diploid cotton species, whose progenitors were putative contributors of the diploid A- and D-subgenomes to tetraploid cotton. Paradoxically, although the genome of *G. arboreum* was about twice the size of that of *G. raimondii*, the total SSR number did not differ by two fold; the SSR frequency was much higher in *G. raimondii* than in *G. arboreum* (190.08 vs 131.03 SSRs/Mb). Previous studies have shown that *gypsy*-like retrotransposon elements have undergone a massive proliferation in larger genomes, like the A-genome, accounting for a major portion of genome-size change [66]. Our recent study on the genome of *G. arboreum* showed that this species did not double its genome size by whole genome duplication but by repetitive DNA amplification, mainly via long-terminal-repeat (LTR) retrotransposons [data not shown]. This result indicated that most microsatellites did not reside in LTRs in the ancestral genome of cotton but in regions of low-copy DNA. Because the genome of *G. arboreum* increased rapidly in size by LTR amplification, the replication slippage events that are thought to produce microsatellites [67] may have occurred relatively more slowly, resulting in the apparent paradox. Given the lack of a whole genome duplication events in *G. arboreum*, the higher frequency of SSRs in *G. raimondii* was consistent with the previous report that a higher density of microsatellites is associated with species in which the genome expanded less recently [68].

In general, the SSR motifs were strongly biased towards AT-rich sequences. For example, the number of AT repeats was 12.6 fold higher than the second most abundant dinucleotide motif (AG), 17.2 fold higher than the third most abundant dinucleotide motif (AC), and 7,771 fold hogher than CG motif. AT-rich motifs showed striking differences in frequency between *G. arboreum* and *G. raimondii* [Additional file 4]. For example, the number of AT motifs was 23470 in *G. raimondii* and 38855 in *G. arboreum*, but AG and AC motifs did not show significant differences, e.g., AG had 2966 and 3084 copies, respectively. Similarly, the number of AAT trinucleotide motifs was 11992 in *G. raimondii* and 20228 in *G. arboreum*. However, AAG and ATC, the second and third most abundant trinucleotides, did not differ significantly in number, e.g., ATC had 1656 and 1865 copies, respectively. Some motifs were even less frequent in *G. arboreum*, such as the fourth-most abundant trinucleotide AAC (1131 and 866 copies, respectively). Thus, AT-rich motifs were the most abundant in both genomes and they accounted for a major part of the excess of SSRs in *G. arboreum*.

### Chromosome-specific SSRs and homologous chromosomes

Many SSR markers have been developed from genomic or EST sequences in cotton [28,29,54,69-71] and used successfully to construct genetic maps and for gene mapping. However, many of them map to at least two positions in tetraploids, mainly because tetraploid cotton (n = 2x = 26, AD) was derived from two diploid species [72], and there are 13 pairs of homologous chromosomes in tetraploid cotton. Distinguishing which chromosome of a pair a given linkage group belongs to is difficult. Our study tested the ability of traditional markers to distinguish homologous chromosomes. We selected 124 and 129 markers from chr07 and chr16, respectively. BLAST analysis showed that 64 of 124 chr07 markers could be detected on chr01 of *G. arboreum*, and 61 of 124 markers could be detected on chr01 of *G. raimondii*; 45 of those detectable markers were shared. Eighty of the 129 chr16 markers could be detected on chr01 of *G. raimondii*, and 59 of 124 markers could be detected in *G. arboreum*, with 53 markers shared. Thus, most traditional markers were located on both homologous chromosomes in tetraploid cotton, although some only mapped to one chromosome.

To make associations between chromosomes and their genetic linkage groups, Wang et al. [42,43] developed a set of chromosome-specific BAC clones to identify all 26 chromosomes. However, FISH is time-consuming and labor-intensive, and BAC clones used as cytogenetic markers could not be easily used as molecular markers in genetic maps. This study benefitted from the genome sequence of two diploid cottons, and a set of chromosome-specific SSRs was developed to identify 26 chromosomes by comparing SSR flanking sequences. Diploid cottons were used to represent the subgenomes in tetraploid cotton based on the high co-linearity between diploid and tetraploid cottons [20-23,25]. We also constructed two linkage groups for two homologous chromosomes in which putative chromosome-specific SSRs were exactly mapped to their homologous chromosomes.

17 of 22 polymorphic SSRs designed from *G. arboreum* mapped to chr07, and 19 of 23 SSRs designed from *G. raimondii* mapped to chr16. A-subgenomes in tetraploid cotton are reported to have longer genetic distances than D-subgenomes [27-32]; this may be why more SSRs mapped to chr16 than to chr07. Nine markers designed in this study did not map to corresponding chromosomes, this is likely a result of errors occurring due to mismatching of chromosomes between diploid and tetraploid cottons. The use of high-density linkage groups of homologous chromosomes, constructed using chromosome-specific SSRs, would allow more SSRs to be mapped. The chromosome-specific SSRs were developed using diploid cotton sequences, and although the co-linearity between diploid and tetraploid cottons is obvious [20-23,25] many SSRs may have changed chromosome location during polyploidization and subsequent genome evolution. Furthermore, the occurrence of misassembles in the cotton genome, especially the A genome (due to its high content of repeats), may have had an impact on our findings. Therefore, a single chromosome-specific SSR cannot represent the entire chromosome. We suggest that a linkage group containing at least three chromosome-specific SSRs will be indicative of a corresponding chromosome.

### High polymorphism of selected SSRs between upland and sealand cottons

High polymorphism is an important character for markers used in high-density genetic map construction and fine mapping of target genes. There are two possible mechanisms for microsatellite evolution: slippage of the DNA polymerase and unequal crossing-over. The first mechanism, which generate gains or losses of one or a few repeat unit(s), accounts for most microsatellite mutations [73,74]. Polymerase slippage rates are highest in dinucleotides, followed by tri- and tetranucleotides, as illustrated in human, mouse, fruit fly, and yeast [75,76]. There are significant positive relationships between repeat length and mutation rate in human [77], fruit fly [78], and yeast [75] microsatellites. In this study, all chromosome-specific SSRs of each motif were ranked by size. The longest 500 SSRs from chr01 of *G. arboreum* and from chr01 of *G. raimondii* were selected from the dinucleotide AT-rich SSRs. Primer pairs were successfully designed to recover high polymorphism. However, two upland cotton varieties, TM-1 and T586, had only 4% polymorphism. Given that SSRs are not highly polymorphic (~5%) among or within *G. hirsutum* cultivars [33-38], this polymorphism rate was reasonable. The near-isogenic relationship of TM-1 and T586 might also limit the polymorphisms recovered by these selected SSRs; T586 was derived from TM-1 by several generation of backcrossing [17].

Several studies have suggested that there is a high frequency of SSR polymorphisms between *G. hirsutum* and *G. barbadense* [20,27-32]. Han et al. [69] detected 18.2% polymorphism for EST–SSRs between these two species, and Yu et al. [61] observed 19.1% polymorphism. Guo et al. [28] and Yu et al. [31] detected 23.9% and 25.0% polymorphism, respectively, between *G. hirsutum* and *G. barbadense* using SSR markers derived from genomic DNA. Similarly, Yu et al. [30] showed 32.9% polymorphism between CRI 36 (*G. hirsutum*) and Hai7124 (*G. barbadense*). The discrepancy in polymorphism rates may be due to different genetic distances between cotton varieties, different kinds of SSRs, or the number of SSRs used. In this study, there was a 52% polymorphism rate between *G. hirsutum* and *G. barbadense*, and a 10% polymorphic rate within *G. hirsutum* for the selected chromosome-specific SSRs. However, a 40% polymorphism rate was observed between *G. hirsutum* and *G. barbadense*, and a 6% rate within *G. hirsutum* when using previously published markers. It seems that the selected SSRs are more polymorphic than normal markers. Therefore, candidate SSRs with the highest polymorphism from this set can be chosen for map construction or gene mapping.

### Use of chromosome-specific SSR resources for cotton research

Chromosome-specific SSRs are efficient tools for chromosome identification, gene location, genetic mapping, QTL tracking, and marker-assisted breeding of cotton. Their greatest advantage is their chromosome specificity derived from the flanking sequences unique to individual chromosomes. This character is very useful in anchoring linkage groups to chromosomes during genetic mapping and in mapping of qualitative-trait genes and in QTL tracking. In general, the number of linkage groups in genetic maps is always more than the number of chromosomes in the genomes, and these linkage groups are often assigned to chromosomes based on chromosome-anchored markers by comparison to previous maps [20,27,29,31,59]. Many linkage groups, especially small groups with few markers, are not assigned to chromosomes, because no marker anchors the group to any particular chromosome. However, genetic maps constructed using chromosome-specific SSRs could solve this problem, because even the smallest linkage groups contain chromosome-specific markers that could be used to assign them to chromosomes. Furthermore, the chromosome-specific SSRs can also be used for gene mapping.

The chromosome-specific SSRs were not clustered in specific regions but distributed evenly across all the chromosomes, so they can be widely used in fingerprinting, genetic diversity analyses, and other applications. To make the chromosome-specific SSRs more user friendly, they were sorted by motif type and repeat length (Additional files 1 and 2). Researchers can choose the most polymorphic candidate SSRs according to position and personal preference. The chromosome-specific SSRs also have shortcomings. They were developed from diploid species based on high co-linearity between diploid and tetraploid cottons assuming no chromosome rearrangements. Some may not chromosome-specific or may not exist on the expected chromosomes in tetraploid cottons. However, multiple chromosome-specific SSRs in the same linkage group could solve this problem. Another issue is that the chromosome-specific SSRs are not robust for comparative evolutionary analyses among chromosomes because similar SSRs may not be found on other chromosomes, but they are efficient tools for comparing different genomes because almost no noise could be detected across chromosomes.

Finally, recent progress in genome sequencing in many species has rapidly advanced map-based cloning and marker-assisted breeding [79-81]. However, because of the scarcity of highly-polymorphic, chromosome-specific molecular markers in cotton, no gene has been cloned through a map-based strategy. The chromosomal loci of many economically-important genes are yet unknown to [82], so marker-assisted breeding in cotton is still underdeveloped relative to some other crops. In the present study, chromosome-specific SSRs for each chromosome were developed that could distinguish homologous chromosomes in tetraploid cotton. The candidate SSRs had high polymorphism, and their exact positions were listed. This very large set of chromosome-specific SSRs, distributed evenly throughout the chromosomes, provides an invaluable resource for cotton genome researches, and it will facilitate the construction of high-resolution maps for positional gene-cloning and marker-assisted breeding.

## Conclusions

The current work characterized microsatellites in cotton and developed chromosome-specific SSRs for map construction and gene mapping. A total of 200,744 and 142,409 SSRs were detected on the 13 chromosomes of *G. arboreum* and *G. raimondii*, respectively. Pentanucleotides were the most common SSR type in both *G. arboreum* and *G. raimondii*. No major differences were observed between the two species except a higher density of SSRs in *G. raimondii*. Chromosome-specific SSRs were obtained by comparing SSR sequences from each chromosome with those from the other 25 chromosomes. There was an average of 7,996 SSRs per chromosome. We proved that the chromosome-specific SSRs could distinguish homologous chromosomes in genetic linkage maps of tetraploid cotton. No crossing of markers between the two chromosomes was observed. In addition, for convenience, all of the SSRs were sorted by motif type and repeat length for each chromosome. These SSRs will facilitate a number of genetic and genomic studies in cotton, including anchoring linkage groups to genetic maps, positional gene-cloning, fingerprinting, and genetic diversity and comparative evolutionary analyses among *Gossypium* species.

## Methods

### Sources of chromosome sequence and materials

The diploid progenitors of *G. arboreum* and *G. raimondii* are putative contributors of the A- and D-subgenomes to tetraploid cottons. The 13 chromosome sequences of *G. arboreum* were downloaded from http://cgp.genomics.org.cn/ [47]. The 13 chromosome sequences of *G. raimondii* were downloaded from the National Center for Biotechnology Information (http://www.ncbi.nlm.nih.gov) [46] for its longer assembled chromosome size.

The mapping population was composed of 184 BC_1_ plants developed from a cross between two upland cotton cultivars, the widely used genetic standard line TM-1 and the multiple dominant gene line T586. The parents TM-1 and T586 were planted and crossed at Anyang, Henan Province, China, in 2012. F_1_ seeds were planted in Hainan Province in winter, 2012. The BC_1_ population was planted at Anyang in 2013. Morphological markers were scored for the presence of the dominant allele of T586 in the BC_1_ population. Screening for polymorphisms using chromosome-specific and previously-published SSRs was conducted using three different cultivars, including two upland cottons (TM-1 and Liaomian 1) and one sealand cotton (Hai 7124).

### Detection of SSRs and primer design

The chromosome sequences were searched for microsatellites with a basic motif of 1–6 bp using the program software SciRoKo 3.4 (SSR Classification and Investigation by Robert Kofler) [48]. The parameters were a minimum total length of 15 and at least three repeats (in the mismatched and fixed penalty search mode). Standardized motifs in SciRoKo were used to represent all variants on both strands of the DNA sequence (e.g., AG includes GA and the reverse complements CT and TC) for consistency in estimating repeat frequencies. Thus, there were two standardized motifs for mononucleotide repeats, four for dinucleotide repeats, 10 for trinucleotide repeats, 33 for tetranucleotide repeats, 102 for pentanucleotide repeats, and 350 for hexanucleotide repeats. SSR content was expressed as ‘number of SSRs per million base pairs’ or as relative frequencies (%) within a particular dataset.

The positions of the SSRs on the chromosomes were recorded, and the flanking sequences were extracted using SciRoKo [48]. Primers pairs were designed from the flanking sequences using Primer3 software [83]. The main parameters for primer design were as follows: primer length 17–27 bp (optimum, 20 bp), PCR product sizes of 125–250 bp, annealing temperature of 60°C, and GC content of 20–80% (optimum, 50%), Other parameters used the program default values.

### Developing of chromosome-specific SSRs

Each chromosome was searched individually for microsatellites. The 200-bp 5′ and 3′ flanking sequences coupled with SSRs were compared for similarity with other such sequences using BLAST (E-value, 1e − 100; we also tried 1e − 10 to 1e − 150. Almost no chromosome-specific SSRs could be detected at 1e − 10 or even 1e − 50. With an E-value of 1e − 150, there were only a few similar SSR flanking sequences. Ultimately, 1e − 100 was determined to be the best E-value for flanking regions of about 400 bp. To detect whether chromosomes were dominated by a small number of SSR types, we first compared all SSR sequences within each chromosome. Then, SSR sequences from each chromosome were compared with those from the other 25 chromosomes to find and remove similar SSRs using Perl scripts; the remaining SSRs with no similar sequences on other chromosomes were considered to be chromosome-specific (Additional files 7, 8, 9 and 10).

To check which chromosomes in diploid cottons corresponded to chr07 and chr16 in tetraploid cotton, 124 and 129 marker sequences from chr07 and chr16 were downloaded (Additional file 11). BLAST analysis showed that 64 of the 124 chr07 markers could be detected on chr01 of *G. arboreum*, and 80 of the 129 chr16 markers could be detected on chr01 of *G. raimondii*. Only about five markers were detected on other chromosomes, indicating that chr07 corresponded to chr01 of *G. arboreum* and chr16 to chr01 of *G. raimondii*. Moreover, 61 markers of chr07 could be detected on chr01 of *G. raimondii*, and 59 markers of chr16 were found on chr01 of *G. arboreum*, which proved that chr01 of *G. arboreum* and chr01 of *G. raimondii* were homologous. Our synteny analysis between the two diploid genomes and genetic maps of tetraploid cotton also showed that tetraploid chr07 corresponded to chr01 of *G. arboreum* and tetraploid chr16 to chr01 of *G. raimondii* (data not shown).

### Marker analysis

Genomic DNA was extracted from young leaves of 184 BC_1_ lines, two parents, and F_1_ plants by the CTAB method described by Paterson et al. [84]. All chromosome-specific SSRs were sorted by motif type and repeat length for each chromosome (Additional files 1 and 2). Five hundred SSR primer pairs each were selected from chr01 of *G. arboreum* and chr01 of *G. raimondii* for the longest dinucleotide AT repeats. Totals of 459 and 448 primer pairs, respectively, were successfully designed, and 124 primer pairs from chr07 and 129 from chr16 were downloaded from www.cottongen.org and www.cottonmarker.org, respectively. After removing redundant SSRs that mapped to both chr07 and chr16, 211 markers were obtained. All SSR primer pairs were synthesized by GenScript (Nanjing, China).

PCR was performed in reaction volumes of 10 μL containing 20 ng template DNA, 0.5 μL 2.5 mM dNTPs, 1× PCR buffer, 0.1 μL 5 U/μL Tap DNA Polymerase, and 0.2 μL of each 10-μΜ primer. The amplification profile was: 5 min at 94°C; 27 cycles of 30 s at 94°C, 30 s at 55°C, 1 min at 72°C, and a final cycle of 5 min at 72°C. The PCR products were separated in 8% polyacrylamide gels.

### Map construction

The SSR primer pairs were first used to screen for polymorphisms between TM-1 and T586. Markers found to be polymorphic were then used to survey 184 individuals of the BC_1_ mapping population. Linkage analysis was conducted using JoinMap3.0 [85] with an LOD score of 6.0. Recombination frequencies were convert to map distances with Kosambi map function [86]. Previously-reported chromosome-anchored SSRs [28,30,54-59] and morphological markers [17] were used to assign the linkage groups to chromosomes. Chromosome nomenclature was consistent with the previous naming system [20].

## Additional files

Additional file 1:
**Thirteen tables that present the exact position and total length of each SSR motif on every chromosome of** ***Gossypium arboreum.***
Additional file 2:
**Thirteen tables that present the exact position and total length of each SSR motif on every chromosome of** ***Gossypium raimondii***. Additional file 3:
**Microsoft Excel file for Supplemental** **Tables S3a–c. Table S3a.** Statistics for all SSRs for each chromosome in *Gossypium arboreum*. Table S3b. Statistics for all SSRs on each chromosome in *Gossypium raimondii*. Table S3c. Summary of SSR density on chromosomes of *Gossypium arboreum* and *Gossypium raimondii*. Additional file 4: Table S4. Distribution of mono-, di-, tri-, tetra-, penta- and hexanucleotide repeats in genomic sequences of *Gossypium arboreum* and *Gossypium raimondii*. Additional file 5: Table S5. Repetition of internal SSRs within each chromosome. Additional file 6: Table S6. Development of chromosome-specific SSRs by comparative analysis. Additional file 7:
**Microsoft Excel file with 13 tables presenting the exact positions and total lengths of all chromosome-specific SSRs on every chromosome of** ***Gossypium arboreum.***
Additional file 8:
**Microsoft Excel file with 13 tables presenting the exact positions and total lengths of all chromosome-specific SSRs on every chromosome of** ***Gossypium raimondii.***
Additional file 9:
**Microsoft Excel file with 13 tables presenting the primers for all chromosome-specific SSRs on every chromosome of** ***Gossypium arboreum.***
Additional file 10:
**Microsoft Excel file with 13 tables presenting the primers for all chromosome-specific SSRs on every chromosome of** ***Gossypium raimondii.***
Additional file 11:
**Microsoft Excel file of Tables S11a and S11b.** **Table S11a.** Sequences of previously-published SSR markers on chr07 in tetraploid cotton. **Table S11b.** Sequences of previously-published SSR markers on chr16 in tetraploid cotton.

## Abbreviations

SSR: Simple sequence repeat
QTL: Quantitative trait locus
MAS: Marker-assisted selection
LG: Linkage group
LTR: Long-terminal-repeat
BAC: Bacterial artificial chromosome
FISH: Fluorescence in situ hybridization
